# Supplementary material for: Bifidobacterium longum subsp. longum BG-L47 boosts growth and activity of Limosilactobacillus reuteri DSM 17938 and its extracellular membrane vesicles
Source: Appl Environ Microbiol. 2024 Jun 18;90(7):e00247-24. doi: 10.1128/aem.00247-24 (PMC11267924; doi:10.1128/aem.00247-24)
Supplement: Supplemental material — Tables S1 to S16; Figures S1 to S3. [file aem.00247-24-s0001.docx]

# Supplementary material

Supplementary table 1: Table showing exclusion criteria in the initial screening of seven bifidobacterial strains.

| **Strain** | **Species** | **Exclusion criteria** | **Weaknesses** | **Strengths** |
| --- | --- | --- | --- | --- |
| 3 | *B. longum* | MIC values ˃breakpoints | n/a | n/a |
| 4 | *B. breve* | MIC values ˃breakpoints | n/a | n/a |
| BG-L48 | *B. longum* | n/a | Bile tolerance, growth variability, weak mucus adhesion | Can grow at low pH, HMO utilization |
| 11 | *B. longum* | Sensitivity to different stress conditions and freeze drying | Very difficult to pelletize | n/a |
| BG-L47 | *B. longum* | n/a | n/a | Fiber utilization, bile tolerance, mucus adhesion, cocultivation with DSM17938 |
| 20 | *B. longum* | Weak growth | n/a | n/a |
| 22 | *B. longum* | Weak growth | n/a | n/a |

Supplementary Table 2: Recipe for Simulated Intestinal Medium (SIM).

| **Simulated intestinal medium (per liter)** |
| --- |
| 2.0 g tryptone (Oxoid) |
| 2.0 g yeast extract |
| 1.0 g NaCl |
| 0.5 g K_2_HPO_4_ |
| 0.5 g KH_2_PO_4_ |
| 0.10 g MgSO_4_ x 7 H_2_O |
| 0.01 g CaCl_2_ x 2 H_2_O |
| 1.0 ml Tween 80 |
| 0.4 g Cysteine-HCl |
| 0.5 g bile (porcine; Sigma B8631) |
| 0.005 g FeSO_4_ x 7 H_2_O |
| 0.05 g MnSO_4_ |
| 100 ng CoCl_2_ x 6 H_2_O (100 μg/ml, 1 ml) |
|  |
| pH was adjusted to 6.8; Autoclaved at 121°C for 15 min |
| Sterile filtered sugar and electron acceptor (when needed) solutions were added before inoculation. Final concentrations: 15 mM of each. |
| Sugar: Glucose.  Electron acceptor: Fructose. |

Supplementary table 3: Start OD of co-cultures

|  | **Start OD** | |
| --- | --- | --- |
|  | **Replicate 1** | **Replicate 2** |
| BG-L47-4% | 0.14 | 0.15 |
| BG-L47-10% | 0.20 | 0.29 |
| BG- L47-25% | 0.74 | 0.70 |
|  |  |  |
| BG-L48- 4% | 0.08 | 0.1 |
| BG-L48-10% | 0.24 | 0.22 |
| BG-L48-25% | 0.51 | 0.56 |
|  |  |  |
| BB536-4% | 0.22 | 0.16 |
| BB536-10% | 0.32 | 0.3 |
| BB536-25% | 0.72 | 0.76 |

Supplementary table 4: Absence of growth of B. longum in Simulated Intestinal Medium.

| **Strain** | **Start CFU/ml (MRS)** | **CFU/ml after 48h in SIM** | | |
| --- | --- | --- | --- | --- |
|  |  | **4%** | **10%** | **25%** |
| BG-L47 | 1.5x10^9^ | 3.0x10^5^ | 3.0x10^6^ | 4.5x10^5^ |
| BG-L48 | 1.4x10^9^ | 1.5x10^6^ | 7.7x10^5^ | 1.7x10^6^ |
| BB536 | 1.0x10^9^ | 5.5x10^5^ | 1.5x10^5^ | 1.5x10^5^ |

Supplementary Table 5: Acetate production by L. reuteri co-incubated with B. longum BG-L47.

| **Sample** | **Acetate (g/l)** |
| --- | --- |
| SIM | - |
| SIM preincubated with BG-L47 | 0.0879614 |
| DSM 17938 grown in SIM preincubated with BG-L47 | 0.845816 |

Supplementary Table 6: Antibiotic resistance profiles of the B. longum strains. Not defined is denoted as ND.

| Antibiotic | BG-L47 | BG-L48 | EFSA cut-off values (mg/L) |
| --- | --- | --- | --- |
| Gentamycin | 32 | 32 | 64 |
| Kanamycin | 256 | 256 | ND |
| Streptomycin | 32 | 16 | 128 |
| Neomycin | 64 | 64 | ND |
| Tetracycline | 1 | 8 | 8 |
| Erythromycin | 0.12 | 0.03 | 1 |
| Clindamycin | 0.12 | 0.06 | 1 |
| Chloramphenicol | 2 | 2 | 4 |
| Ampicillin | 0.12 | 0.12 | 2 |
| Penicillin | 0.25 | 0.12 | ND |
| Vancomycin | 2 | 0.5 | 2 |
| Quinupristin-  Dalfopristin | 0.25 | 0.25 | ND |
| Linezolid | 1 | 1 | ND |
| Trimethoprim | 16 | 16 | ND |
| Ciprofloxacin | 8 | 8 | ND |
| Rifampicin | 0.5 | 0.25 | ND |


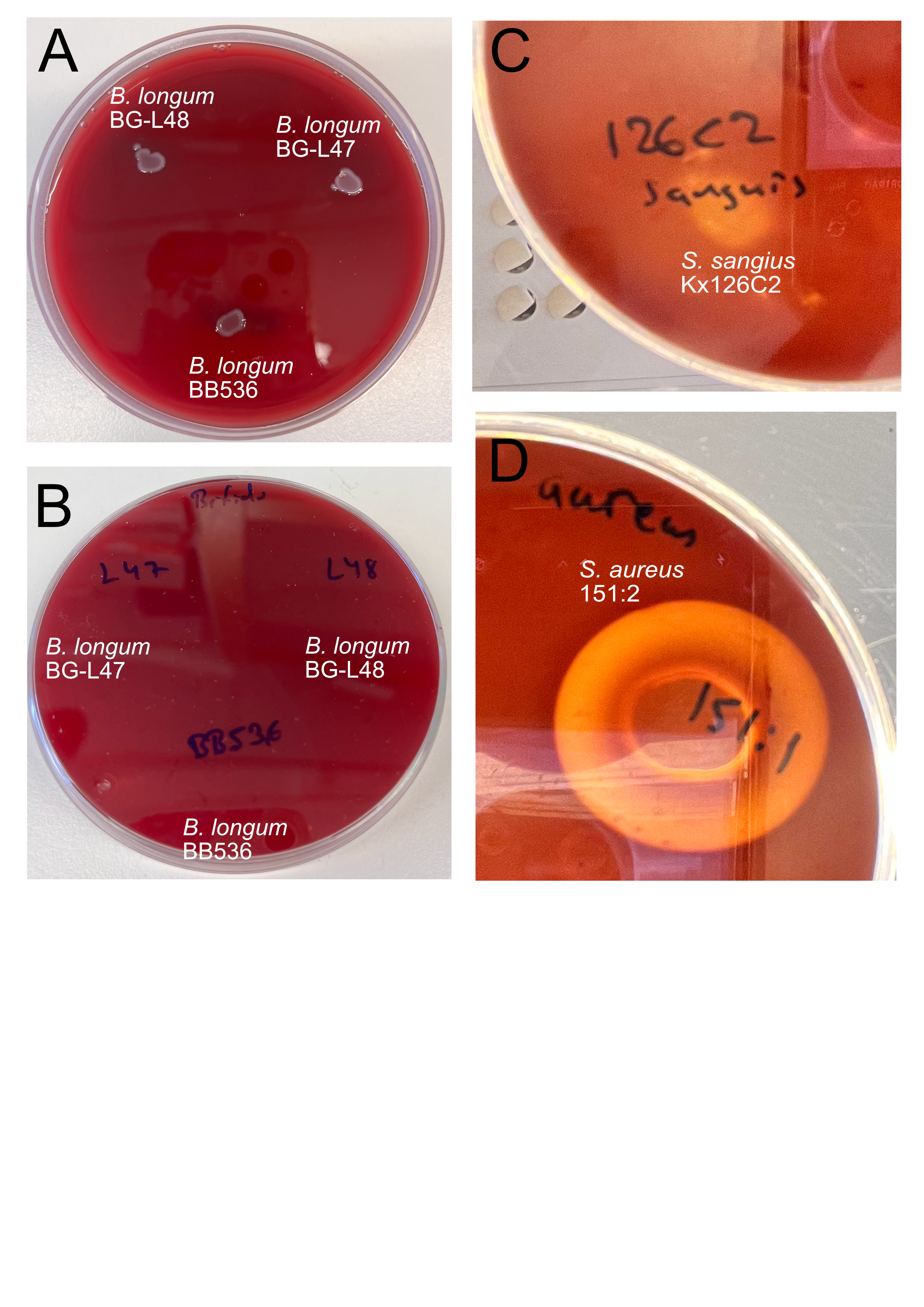


Supplementary Figure 1: Hemolytic activity of BG-L47, BG-L48, and BB536. (A) shows the colonies of BG-L47, BG-L48 and BB536. (B) shows plate (A) from underneath. (C) shows S. sanguis Kx126C2 (positive control for alpha hemolysis). (D) shows S. aureus 151:2 (positive control for beta hemolysis).

Supplementary Table 7: Genome characteristics

|  | **BG-L47** |
| --- | --- |
| Assembly level | Complete genome |
| Number of contigs | 1 |
| Size (bp) | 2,378,960 |
| GC content (%) | 60.1 |
| CDS | 1941 |
| ANI vs *B. longum* subsp. *longum* ATCC 15707 | 98.13% |

Supplementary Table 8: Number of genes in the genomes of BG-L47 and BB536 in different COG classes.

|  |  | **BG-L47** | **BB536** |
| --- | --- | --- | --- |
|  | **COG class** |  |  |
| INFORMATION STORAGE AND PROCESSING | J - Translation, ribosomal structure and biogenesis | 138 | 137 |
|  | A – RNA processing and modification | 0 | 0 |
|  | K - Transcription | 121 | 128 |
|  | L - Replication, recombination and repair | 139 | 136 |
| CELLULAR PROCESSES AND SIGNALING | D - Cell cycle control, cell division, chromosome partitioning | 31 | 28 |
|  | V - Defense mechanisms | 53 | 58 |
|  | T - Signal transduction mechanisms | 35 | 37 |
|  | M - Cell wall/membrane/envelope biogenesis | 76 | 80 |
|  | N - Cell motility | 1 | 2 |
|  | U - Intracellular trafficking, secretion, and vesicular transport | 45 | 40 |
|  | O - Posttranslational modification, protein turnover, chaperones | 42 | 44 |
| METABOLISM | C - Energy production and conversion | 56 | 50 |
|  | G - Carbohydrate transport and metabolism | 157 | 146 |
|  | E - Amino acid transport and metabolism | 133 | 129 |
|  | F - Nucleotide transport and metabolism | 75 | 71 |
|  | H - Coenzyme transport and metabolism | 63 | 62 |
|  | I - Lipid transport and metabolism | 38 | 38 |
|  | P - Inorganic ion transport and metabolism | 66 | 63 |
|  | Q - Secondary metabolites biosynthesis, transport and catabolism | 6 | 7 |
| POORLY CHARACTERIZED | S - Function unknown | 290 | 300 |
| TOTAL |  | 1565 | 1556 |

Supplementary table 9: Production of biogenic amines and lactate isomers by BG-L47.

| **Strain** | **Cultivation-medium** | **Histamine** | **Tyramine** | **Putrescine** | **Cadaverine** | **D-lactate** | **L-lactate** |
| --- | --- | --- | --- | --- | --- | --- | --- |
| BG-L47 | MRS broth | < 130 ng/ml | < 110 ng/ml | < 440 ng/ml | < 250 ng/ml | 0 mg/ml | 6.6 mg/ml |
| BG-L47 | Enriched MRS broth | < 130 ng/ml | < 110 ng/ml | < 440 ng/ml | < 250 ng/ml |  |  |

Supplementary Table 10: Genomic Islands present in the genomes of BG-L47 and BB536

| **BG-L47** | | | | **BB536** | | | |
| --- | --- | --- | --- | --- | --- | --- | --- |
| **Island start** | **Island end** | **Length** | **Antibiotic resistance/ Virulence factors** | **Island start** | **Island end** | **Length** | **Antibiotic resistance/ Virulence factors** |
| 16985 | 21257 | 4272 | - | 17208 | 31365 | 14157 | - |
| 33694 | 45183 | 11489 | - | 43461 | 54938 | 11477 | - |
| 35739 | 45344 | 9605 | - | 45406 | 55208 | 9802 | - |
| 86778 | 95504 | 8726 | - | 96570 | 102447 | 5877 | - |
| 124741 | 130188 | 5447 | - | 258899 | 266725 | 7826 | - |
| 171269 | 175928 | 4659 | - | 266943 | 276272 | 9329 | - |
| 235635 | 240116 | 4481 | - | 315786 | 342618 | 26832 | - |
| 280293 | 297824 | 17531 | - | 327255 | 334202 | 6947 | - |
| 484935 | 503362 | 18427 | - | 444741 | 451654 | 6913 | - |
| 487619 | 505811 | 18192 | - | 448498 | 454293 | 5795 | - |
| 709313 | 714491 | 5178 | - | 510124 | 525506 | 15382 | - |
| 826587 | 916997 | 90410 | - | 735697 | 740046 | 4349 | - |
| 827466 | 838487 | 11021 | - | 876553 | 883587 | 7034 | - |
| 828207 | 839299 | 11092 | - | 876678 | 883085 | 6407 | - |
| 839478 | 879856 | 40378 | - | 965839 | 973703 | 7864 | - |
| 839686 | 845382 | 5696 | - | 1059312 | 1076174 | 16862 | - |
| 870796 | 880828 | 10032 | - | 1067437 | 1076749 | 9312 | - |
| 881401 | 890838 | 9437 | - | 1102108 | 1107946 | 5838 | - |
| 888677 | 916997 | 28320 | - | 1123348 | 1136662 | 13314 | - |
| 899134 | 906095 | 6961 | - | 1148568 | 1230459 | 81891 | - |
| 910245 | 917308 | 7063 | - | 1149332 | 1165230 | 15898 | - |
| 948976 | 956029 | 7053 | - | 1154034 | 1178288 | 24254 | - |
| 949107 | 955527 | 6420 | - | 1168262 | 1176048 | 7786 | - |
| 1040347 | 1048523 | 8176 | - | 1176730 | 1180808 | 4078 | - |
| 1135980 | 1141755 | 5775 | - | 1182054 | 1202444 | 20390 | - |
| 1136147 | 1150162 | 14015 | - | 1182073 | 1232935 | 50862 | - |
| 1144472 | 1148820 | 4348 | - | 1216188 | 1224135 | 7947 | - |
| 1181419 | 1190389 | 8970 | - | 1227151 | 1232651 | 5500 | - |
| 1259482 | 1268870 | 9388 | - | 1261634 | 1273371 | 11737 | - |
| 1296536 | 1306384 | 9848 | - | 1276294 | 1280839 | 4545 | - |
| 1311971 | 1353539 | 41568 | - | 1328067 | 1337453 | 9386 | - |
| 1319145 | 1329025 | 9880 | - | 1381437 | 1386111 | 4674 | - |
| 1340355 | 1346920 | 6565 | - | 1574869 | 1591876 | 17007 | - |
| 1349435 | 1355730 | 6295 | - | 1579723 | 1588288 | 8565 | - |
| 1406044 | 1412994 | 6950 | - | 1667461 | 1671642 | 4181 | - |
| 1678223 | 1683429 | 5206 | - | 1707189 | 1711773 | 4584 | - |
| 1753647 | 1762927 | 9280 | - | 1744743 | 1749355 | 4612 | - |
| 1973998 | 1980409 | 6411 | - | 1792373 | 1801514 | 9141 | - |
| 2054251 | 2062814 | 8563 | - | 2066503 | 2076682 | 10179 | - |
| 2158238 | 2167601 | 9363 | - | 2067920 | 2073959 | 6039 | - |
| 2164480 | 2170594 | 6114 | - | 2170274 | 2189795 | 19521 | - |
| 2193478 | 2210164 | 16686 | - | 2178318 | 2186001 | 7683 | - |
| 2244037 | 2248221 | 4184 | - | 2181136 | 2189795 | 8659 | - |
| 2278423 | 2292880 | 14457 | - | 2195922 | 2200477 | 4555 | - |
| 2365798 | 2375989 | 10191 | - | 2216167 | 2232860 | 16693 | - |
|  |  |  |  | 2280596 | 2293276 | 12680 | - |
|  |  |  |  | 2361635 | 2368028 | 6393 | - |
|  |  |  |  | 2406298 | 2416492 | 10194 | - |

Supplementary Figure 2: Alpha diversity at the genus level with Shannon, Simpson and inverted Simpson corrections. Abbreviations are H for high dose, L for low dose, P for placebo, HA for high dose after treatment, HB for high dose before treatment, LA for low dose after treatment, LB for low dose before treatment, PA for placebo after treatment, PB for placebo before treatment.

Supplementary Table 11: Beta diversity at genus level. Abbreviations are HA for high dose after treatment, HB for high dose before treatment, LA for low dose after treatment, LB for low dose before treatment, PA for placebo after treatment, PB for placebo before treatment.

|  | Permutest.pvalue | TukeyHSD.adj.pvalue |
| --- | --- | --- |
| HA-HB | 0.002 | 0.005 |
| HA-LA | 0.503 | 0.963 |
| HA-LB | 0.001 | 0 |
| HA-PA | 0.518 | 0.971 |
| HA-PB | 0.008 | 0.02 |
| HB-LA | 0.004 | 0.051 |
| HB-LB | 0.353 | 0.978 |
| HB-PA | 0.001 | 0 |
| HB-PB | 0.531 | 0.997 |
| LA-LB | 0.001 | 0.006 |
| LA-PA | 0.18 | 0.605 |
| LA-PB | 0.021 | 0.154 |
| LB-PA | 0.001 | 0 |
| LB-PB | 0.176 | 0.833 |
| PA-PB | 0.003 | 0.002 |

Supplementary table 12: Significantly changed genera in among the different groups between day 0 and 28. Green arrow indicates that 4 individuals showed the same trend within the group.

|  | **Low Dose** | **High Dose** | **Placebo** | **Kingdom** | **Phylum** | **Class** | **Order** | **Family** | **Genus** |
| --- | --- | --- | --- | --- | --- | --- | --- | --- | --- |
| ASV176 |  | **↑** |  | Bacteria | Bacteroidota | Bacteroidia | Bacteroidales | Marinifilaceae | Odoribacter |
| ASV341 | **↑** | **↑** |  | Bacteria | Firmicutes | Bacilli | Erysipelotrichales | Erysipelatoclostridiaceae | Erysipelatoclostridiaceae |
| ASV342 |  |  | **↓** | Bacteria | Actinobacteriota | Coriobacteriia | Coriobacteriales |  |  |
| ASV505 | **↓** | **↓** | **↓** | Bacteria | Proteobacteria | Gammaproteobacteria | Enterobacterales |  |  |
| ASV718 |  | **↑** |  | Bacteria | Cyanobacteria | Cyanobacteriia | Chloroplast | Chloroplast | Chloroplast |
| ASV722 |  | **↓** |  | Archaea | Euryarchaeota | Methanobacteria | Methanobacteriales | Methanobacteriaceae | Methanosphaera |
| ASV727 | **↑** | **↑** | **↑** | Bacteria | Proteobacteria | Gammaproteobacteria | Burkholderiales | Comamonadaceae | Comamonas |
| ASV752 | **↓** |  |  | Bacteria | Firmicutes | Clostridia | Lachnospirales | Lachnospiraceae | Lachnospiraceae_NK4B4_group |
| ASV775 |  |  | **↑** | Bacteria | Firmicutes | Bacilli | Lactobacillales | Leuconostocaceae | Weissella |
| ASV784 |  |  | **↑** | Bacteria | Firmicutes | Clostridia | Oscillospirales | Oscillospiraceae | Oscillospiraceae |
| ASV815 |  | **↓↑** | **↓** | Bacteria | Firmicutes | Clostridia | Lachnospirales | Lachnospiraceae | Ruminococcus_gnavus_group |
| ASV864 |  | **↓** |  | Bacteria | Firmicutes | Clostridia | Clostridia | Gracilibacteraceae |  |
| ASV945 | **↓** | **↓** | **↓** | Bacteria | Bacteroidota | Bacteroidia | Bacteroidales | Tannerellaceae | Tannerellaceae |
| ASV1030 | **↓** | **↓** | **↓** | Bacteria | Firmicutes | Bacilli | Erysipelotrichales | Erysipelatoclostridiaceae | Coprobacillus |
| ASV1077 |  | **↓** |  | Bacteria | Proteobacteria | Gammaproteobacteria | Pseudomonadales | Pseudomonadaceae | Pseudomonas |
| ASV1085 | **↓** | **↓** |  | Bacteria | Firmicutes | Clostridia | Lachnospirales | Lachnospiraceae | Frisingicoccus |
| ASV1179 | **↓** |  |  | Bacteria | Gemmatimonadota | Gemmatimonadetes | Gemmatimonadales | Gemmatimonadaceae |  |
| ASV1219 | **↓** | **↓** | **↓** | Bacteria | Actinobacteriota | Coriobacteriia | Coriobacteriales | Coriobacteriales_Incertae_Sedis | Phoenicibacter |
| ASV1235 | **↑** |  |  | Bacteria | Proteobacteria | Alphaproteobacteria | Rickettsiales | Mitochondria | Mitochondria |
| ASV1236 | **↓↑** | **↓↑** | **↓** | Bacteria | Firmicutes | Clostridia | Lachnospirales | Lachnospiraceae | Hungatella |
| ASV1258 | **↓** | **↓** |  | Bacteria | Bacteroidota | Bacteroidia | Bacteroidales | Muribaculaceae |  |
| ASV1318 |  | **↑** | **↓** | Bacteria | Firmicutes | Clostridia | Lachnospirales | Lachnospiraceae | UC5-1-2E3 |
| ASV1324 | **↓↑** | **↑** |  | Bacteria | Proteobacteria | Gammaproteobacteria | Enterobacterales | Hafniaceae | Hafnia-Obesumbacterium |
| ASV1459 |  |  | **↓** | Bacteria | Gemmatimonadota | Gemmatimonadetes | Gemmatimonadales | Gemmatimonadaceae | Gemmatimonas |
| ASV1509 | **↑** |  |  | Bacteria | Firmicutes | Bacilli | Lactobacillales | Carnobacteriaceae | Granulicatella |
| ASV1532 |  | **↓↑** | **↑** | Bacteria | Firmicutes | Clostridia | Lachnospirales | Lachnospiraceae | Catenibacillus |
| ASV1575 |  | **↓** |  | Bacteria | Firmicutes | Clostridia | Lachnospirales | Lachnospiraceae | Anaerostignum |
| ASV1579 | **↓↑** |  |  | Bacteria | Actinobacteriota | Coriobacteriia | Coriobacteriales | Eggerthellaceae | Enteroscipio |
| ASV1641 | **↑** | **↓↑** |  | Bacteria | Actinobacteriota | Actinobacteria | Corynebacteriales | Corynebacteriaceae | Corynebacterium |
| ASV1709 | **↓** | **↓** | **↓** | Bacteria | Firmicutes | Clostridia | Peptostreptococcales-Tissierellales | Peptostreptococcales-Tissierellales | Fenollaria |
| ASV2082 |  |  | **↑** | Bacteria | Firmicutes | Clostridia | Christensenellales | Christensenellaceae | Catabacter |
| ASV2085 |  | **↓** |  | Bacteria | Firmicutes | Bacilli | Erysipelotrichales | Erysipelotrichaceae | Faecalitalea |
| ASV2101 |  | **↓** |  | Bacteria | Firmicutes | Bacilli | Erysipelotrichales | Erysipelotrichaceae | Solobacterium |
| ASV2144 |  | **↓↑** |  | Bacteria | Firmicutes | Clostridia | Eubacteriales | Eubacteriaceae | Eubacterium |
| ASV2326 |  |  | **↓↑** | Bacteria | Proteobacteria | Gammaproteobacteria | Aeromonadales | Succinivibrionaceae | Succinivibrio |
| ASV2368 | **↑** |  | **↓↑** | Bacteria | Actinobacteriota | Actinobacteria | Micrococcales | Micrococcaceae | Rothia |
| ASV2504 | **↑** |  |  | Bacteria | Firmicutes | Clostridia | Peptostreptococcales-Tissierellales | Peptostreptococcaceae | Peptostreptococcus |
| ASV2545 |  | **↑** |  | Bacteria | Firmicutes | Clostridia | Oscillospirales | Ruminococcaceae | Harryflintia |
| ASV2683 |  |  | **↓↑** | Bacteria | Firmicutes | Clostridia | Peptostreptococcales-Tissierellales | Peptostreptococcales-Tissierellales | Anaerococcus |
| ASV2733 |  |  | **↓** | Bacteria | Actinobacteriota | Actinobacteria | Actinomycetales | Actinomycetaceae | Varibaculum |
| ASV3086 |  |  | **↓** | Bacteria | Firmicutes | Clostridia | Peptostreptococcales-Tissierellales | Peptostreptococcales-Tissierellales | Parvimonas |


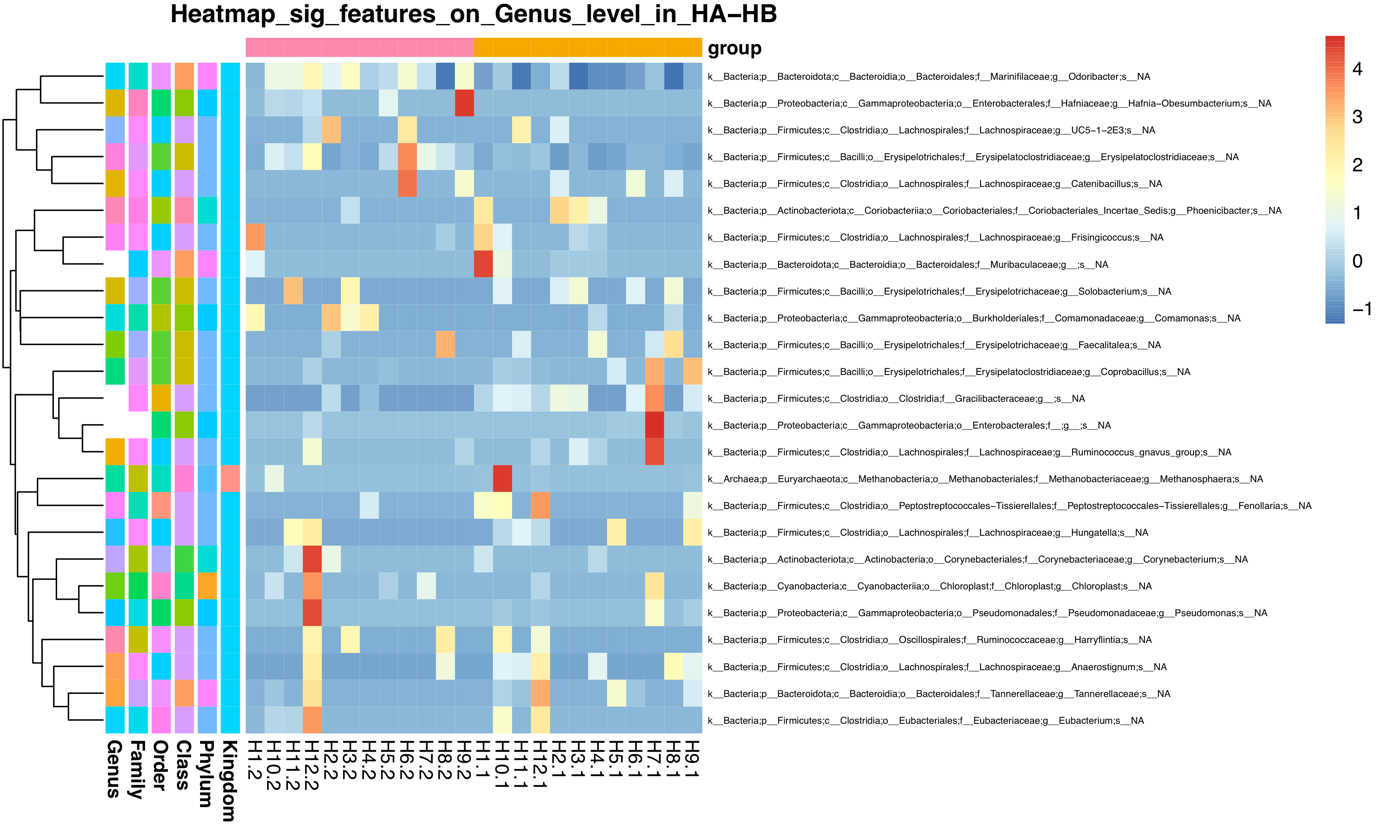


Supplementary Figure 3: Heatmap showing significantly changed bacteria of high-dose group before and after treatment. HX.1 denotes prior to treatment and HX.2 denotes after treatment (X = any number).

Supplementary table 13: API of B. longum BG-L47, BG-L48 and BB536 under aerobic conditions (API tubes covered by oil and incubated aerobically at 37°C). The level of growth was assessed by estimating color changes (scaled 1-5).

|  | **BG-L47** | **BG-L48** | **BB536** |
| --- | --- | --- | --- |
| L-arabinose | 4 | 4 | 5 |
| D-ribose |  | 4 |  |
| D-xylose | 3 |  | 5 |
| D-Galactose | 5 | 5 | 1 |
| D-Glucose | 5 | 5 | 5 |
| D-Fructose | 4 | 1 | 5 |
| D-Sorbitol | 5 |  |  |
| D-Maltose | 1 | 5 | 4 |
| D-Lactose | 1 | 5 | 5 |
| D-Melibiose | 5 | 5 | 1 |
| D-Saccharose |  | 5 |  |
| D-Melezitose | 1 |  |  |
| D-Raffinose | 1 | 5 |  |
| D-Turanose |  |  | 1 |
| Potassium gluconate |  | 3 |  |

Supplementary Table 14: Composition of modified MRS (mMRS) medium

| **mMRS media composition** |
| --- |
| 1% bactopeptone (wt/vol) |
| 0.5% yeast extract (wt/vol) |
| 0.2% dipotassium phosphate (wt/vol) |
| 0.5% sodium acetate (wt/vol) |
| 0.2% ammonium citrate (wt/vol) |
| 0.02% magnesium sulfate (wt/vol) |
| 0.005% manganese sulfate (wt/vol) |
| 0.1% Tween 80 (vol/vol) |
| 0.05% cysteine |
| 1% (w/v) carbon source |

Supplementary Table 15: Inclusion criteria for human safety study

| 1 | Willing and able to give written informed consent for participation in the study. |
| --- | --- |
| 2 | Healthy male or female subject aged 18-65 years inclusive. |
| 3 | Body Mass Index (BMI) ≥ 18.0 and ≤ 32.0 kg/m^2^. |
| 4 | Clinically normal medical history, physical findings, vital signs, and laboratory values at the time of screening, as judged by the Investigator. |
| 5 | Women of child bearing potential (WOCBP) had to practice abstinence (only allowed when this was the preferred and usual lifestyle of the subject) or had to agree to use a highly effective method of contraception with a failure rate of < 1% to prevent pregnancy (combined [oestrogen and progestogen containing] hormonal contraception associated with inhibition of ovulation [oral, intravaginal, transdermal], progestogen-only hormonal contraception associated with inhibition of ovulation [oral, injectable, implantable], intrauterine device [IUD]or intrauterine hormone-releasing system [IUS]) from at least 4 weeks prior to first IP dose to 4 weeks after last dose. Women of non-childbearing potential were defined as pre-menopausal females who were sterilized (tubal ligation or permanent bilateral occlusion of fallopian tubes); or post-menopausal defined as 12 months of amenorrhea (in questionable cases a blood sample with simultaneous detection of follicle stimulating hormone [FSH] 25-140 IE/L is confirmatory). |

Supplementary Table 16: Exclusion criteria for human safety study.

| 1 | History of any clinically significant disease or disorder which, in the opinion of the Investigator, could either put the subject at risk because of participation in the study, or influence the results or the subject’s ability to participate in the study. |
| --- | --- |
| 2 | Any clinically significant illness, medical/surgical procedure, or trauma within 4 weeks of the first administration of IP. |
| 3 | Malignancy within the past 5 years with the exception of in situ removal of basal cell carcinoma. |
| 4 | Any planned major surgery within the duration of the study. |
| 5 | Any positive result on screening for serum hepatitis B surface antigen, hepatitis C antibodies or HIV. |
| 6 | History of severe allergy/hypersensitivity or ongoing allergy/hypersensitivity, as judged by the Investigator, or history of hypersensitivity to *Bifidobacterium* probiotic treatment. |
| 7 | History of, or ongoing GI disorder, including but not limited to irritable bowel syndrome (IBS), constipation, loose stools, or excess gas which, in the discretion of the Investigator, could influence the results or the subject’s ability to participate in the study. |
| 8 | Lactose intolerance (that in the opinion of the Investigator could interfere with the use of milk drinks once daily for 28 days). |
| 9 | Regular use of any prescribed or non-prescribed medication including antacids and analgesics within 2 weeks prior to the first administration of IP, at the discretion of the Investigator. |
| 10 | Any use of antibiotics (except local treatment, e.g., eye drops) within two weeks prior to the first administration of IP. |
| 11 | Planned treatment or treatment with an investigational drug within 3 months prior to Day 1. Subjects consented and screened but not dosed in previous phase I studies were not excluded. |
| 12 | Current smokers or users of nicotine products. Irregular use of nicotine (e.g., smoking, snuffing, chewing tobacco) less than three times per week was allowed before screening visit. |
| 13 | Positive screen for drugs of abuse or alcohol at screening or on admission to the unit prior to administration of the IP. |
| 14 | History of alcohol abuse or excessive intake of alcohol, as judged by the Investigator. |
| 15 | Presence or history of drug abuse, as judged by the Investigator. |
| 16 | History of, or current use of, anabolic steroids, as judged by the Investigator. |
| 17 | Excessive caffeine consumption defined by a daily intake of >5 cups of caffeine. |
| 18 | Plasma donation within one month of screening or blood donation (or corresponding blood loss) during the three months prior to screening. |
| 19 | Investigator considers the subject unlikely to comply with study procedures, restrictions, and requirements. |
